# Changes in the Number and Morphology of Dendritic Spines in the Hippocampus and Prefrontal Cortex of the C58/J Mouse Model of Autism

**DOI:** 10.3389/fncel.2021.726501

**Published:** 2021-09-20

**Authors:** Isabel Barón-Mendoza, Emely Maqueda-Martínez, Mónica Martínez-Marcial, Marisol De la Fuente-Granada, Margarita Gómez-Chavarin, Aliesha González-Arenas

**Affiliations:** ^1^Departamento de Medicina Genómica y Toxicología Ambiental, Instituto de Investigaciones Biomédicas, Universidad Nacional Autónoma de México, Mexico City, Mexico; ^2^Unidad de Modelos Biológicos, Instituto de Investigaciones Biomédicas, Universidad Nacional Autónoma de México, Mexico City, Mexico; ^3^Departamento de Fisiología, Facultad de Medicina, Universidad Nacional Autónoma de México, Mexico City, Mexico

**Keywords:** autism, dendritic spines, structural plasticity, hippocampus, prefrontal cortex, C58/J

## Abstract

Autism spectrum disorder (ASD) has a broad range of neurobiological characteristics, including alterations in dendritic spines, where approximately 90% of excitatory synapses occur. Therefore, changes in their number or morphology would be related to atypical brain communication. The C58/J inbred mouse strain displays low sociability, impaired communication, and stereotyped behavior; hence, it is considered among the animal models suitable for the study of idiopathic autism. Thus, this study aimed to evaluate the dendritic spine differences in the hippocampus and the prefrontal cortex of C58/J mice. We found changes in the number of spines and morphology in a brain region-dependent manner: a subtle decrease in spine density in the prefrontal cortex, higher frequency of immature phenotype spines characterized by filopodia-like length or small morphology, and a lower number of mature phenotype spines with mushroom-like or wide heads in the hippocampus. Moreover, an *in silico* analysis showed single nucleotide polymorphisms (SNPs) at genes collectively involved in regulating structural plasticity with a likely association with ASD, including MAP1A (Microtubule-Associated Protein 1A), GRM7 (Metabotropic Glutamate Receptor, 7), ANKRD11 (Ankyrin Repeat Domain 11), and SLC6A4 (Solute Carrier Family 6, member 4), which might support the relationship between the C58/J strain genome, an autistic-like behavior, and the observed anomalies in the dendritic spines.

## Introduction

Autism spectrum disorder (ASD) refers to a group of neurodevelopmental disorders characterized by persistent deficits in social communication and social interaction across multiple contexts, as well as the manifestation of restricted, repetitive patterns of behavior, interests, or activities (American Psychiatric Association, 2013).

ASD has a broad range of neurobiological characteristics, including structural plasticity-associated changes, such as alterations in dendritic spines (Bernardinelli et al., 2014; Lin et al., 2016; Gilbert and Man, 2017). Hutsler and Zhang (2010) reported the first findings of spine anomalies in post-mortem samples from different Brodmann areas. They observed higher spine density in layer II of parietal (BA 7), frontal (BA 9), and temporal (BA 21) cortex regions, and in layer V, only within the temporal lobe. Moreover, they suggested an increased length of dendritic spines, which might point to the frequent presence of immature shape types (Hutsler and Avino, 2015). Similarly, Fragile X patients, a comorbid syndrome of ASD, exhibited a higher number and longer dendritic spines on layer V of the visual and temporal cortex (Irwin et al., 2001).

The characterization of iPSC (induced Pluripotent Stem Cells)-derived neurons from people with autism carrying SHANK3 (SH3 And Multiple Ankyrin Repeat Domains Protein) *de novo* mutations also showed a decreased spine density as well as lower volume spine heads (Gouder et al., 2019). Additionally, abnormal cytoskeleton dynamics associated with altered Rho-GTPases signaling have been observed in iPSC-derived neuronal cells (Griesi-Oliveira et al., 2018), which might be related to the neuronal structural differences found in people with ASD diagnosis.

Since approximately 90 percent of excitatory synapses locate in dendritic spines, changes in their number and morphology might affect synaptic transmission regulation and neuronal structural plasticity in general (Lefebvre et al., 2015) and could be related to the atypical brain communication displayed by people with ASD (Martínez-Cerdeño, 2017).

Moreover, there is evidence of dendritic spine alterations in murine models associated with ASD, such as the Rett, Fragile X, and 15q11-13-duplication syndrome mouse models (Cruz-Martín et al., 2010; Wang et al., 2017), as well as the CYFIP1 (cytoplasmic FMR1 interacting protein 1), CNTNAP2 (contactin-associated protein-like 2), DLGAP2 (DLG associated protein 2), and SHANK1 (SH3 and multiple ankyrin repeat domains 1) gene-deficient mice (Hung et al., 2008; Jiang-Xie et al., 2014; Pathania et al., 2014; Gdalyahu et al., 2015). Nevertheless, reports focused on studying structural plasticity modifications in mouse models of non-syndromic/idiopathic autism are lacking.

The C58/J inbred mouse strain displays low sociability, impaired communication, and stereotyped behavior, and it is considered among the animal models suitable to study idiopathic autism. It has been reported that these autistic-like mice also have polymorphisms in ASD-associated genes involved in neurotransmission and synaptic function (Moy et al., 2014). Furthermore, according to volumetric analysis, C58/J mice exhibit both enlarged and smaller brain regions that might be related to their stereotyped behavior, which involves vertical hindlimb jumping and backward somersaulting (Wilkes et al., 2020). Changes in the dendritic tree of the prefrontal cortex and the hippocampus of this strain, along with an abnormal expression of cytoskeleton dynamic-related proteins, have also been already observed (Barón-Mendoza et al., 2018, 2019).

Thus, in this study, we aimed to analyze if there were changes in the number and morphology of dendritic spines in the pyramidal neurons from the prefrontal cortex and the hippocampus of the C58/J autistic-like strain and determine if this could be related to polymorphisms at genes involved in cytoskeleton regulation and neuronal structural plasticity, or associated to differences in the expression of regulator proteins of neurite remodeling, such as the brain-derived neurotrophic factor (BDNF).

## Materials and Methods

### Animals

Mice from C57 BL/6J (neurotypical phenotype) and C58/J (autistic-like phenotype) strains were acquired from The Jackson Laboratory (Bay Harbor, ME, United States). Upon arrival at our Institute’s facilities, they showed negative medical tests for bacterium, fungi, and viruses, according to the USDA (United States Department of Agriculture). After arriving from The Jackson Laboratory, mice underwent a 2-week adaptation period to the new housing conditions at the Animal Care Facility Unit of the Instituto de Investigaciones Biomedicas, UNAM.

The period of stay follows two parameters: the first one refers to “The Three Rs principle” (Russell et al., 1959); the second relates to collecting samples for parasitological analysis that determine the duration of the animal’s quarantine. The aforementioned complied with Mexican procedure NOM 062 Z00. Animals were kept for crossbreeding (father and mother) until birth. The litters had four to five pups.

The animals were housed in an individually ventilated caging system under standard conditions (mean temperature 22 ± 2°C, 40 ± 10% relative humidity) on a sterilized wood shavings bedding (Envigo, Indianapolis, IN, United States) and maintained under a reversed 12:12 h light/dark cycle (lights on 19:00–7:00 h). Mice received a commercial pelletized diet (T.G. rodent diet T2018S.15, Envigo), and water from an automated watering system *ad libitum*.

Eight male mice, 10 weeks old, from C58/J (four animals) and C57 BL/6J (four animals) strains were sacrificed by decapitation according to the AVMA Guidelines for the Euthanasia of Animals: 2020 Edition (Leary et al., 2020). All experiments were approved by the local Institutional Animal Care and Research Advisory Committee (CICUAL, ID 189), from the Universidad Nacional Autonoma de Mexico, and complied with the International Guidelines of ethical care and use of animals (Leary et al., 2020).

### Golgi-Cox Staining and Sample Preparation

Brains were removed and rinsed with cold Milli-Q water to wash away the blood and immediately transferred to a Golgi–Cox staining solution.

The FD Rapid GolgiStain kit (FD Neurotechnologies, Columbia, MD, United States) is an improved Golgi-Cox impregnation technique designed based on the principle of the methods described by Ramón-Moliner and Glaser and Van der Loos (Ramón-Moliner, 1970; Glaser and Van der Loos, 1981; Zaqout and Kaindl, 2016). The Golgi–Cox staining procedure includes (1) the impregnation step, which consists of preparing a 5% w/v solution of potassium dichromate, mercuric chloride, and potassium chromate dissolved in Milli-Q water, and (2) the developing step, in which samples are treated with a mixture of 75% ammonia and 1% sodium thiosulfate solutions (Zaqout and Kaindl, 2016). The staining procedure followed the manufacturer’s recommendations.

Each brain was immersed in 3 ml of the impregnation solution prepared with an equal volume mixture of solutions A and B (mercuric chloride, potassium chromate, and potassium dichromate). After 24 h, the solution was replaced with a new mixture of A and B solutions. Brains were stored in darkness for 2 weeks at 25°C. Before tissue sectioning, brains were immersed in a cryoprotectant solution composed of 300 g sucrose, 10 g polyvinylpyrrolidone, and 300 ml ethyl glycol in 500 ml phosphate buffer and stored for another week under the same conditions.

Afterward, the whole brains were frozen, and 70-μm-thick slices (coronal sections) were cut in a cryostat. The slices were mounted in gelatin-coated microscope slides (2.5 g gelatin, 0.2 g Cr2(SO4)3/500 ml).

For the developing step, slices were treated with a mixture of the solutions D and E. Next, the slides were rinsed with Milli-Q water, air dried naturally, and finally protected with Cytoseal resin (Thermo Fisher, Waltham, MA, United States) and coverslipped.

### Image Acquisition and Dendritic Spine Analysis

Twenty well-stained pyramidal neurons from the layer II/III of the prefrontal cortex (bregma 3.17–2.93 mm) and the CA1 region of the dorsal hippocampus (bregma –2.03 to –2.27 mm) (Paxinos and Franklin, 2004) of each strain (five neurons per mouse) were identified and captured in an Olympus BX51-WI-DSU (Disk Scanning Unit) microscope (Olympus, Tokyo, Japan; Objective Microscope: 100×) coupled to the Stereoinvestigator 9^®^ software (MBF Bioscience, Williston, VT, United States).

Series of *z*-stack images were taken (distance between images, 2 μm) for the apical and basal compartment of each neuron. A well-stained neuron shows a complete dendrite filling and well-stained dendritic spines across the dendritic shafts, with no breaks in dendritic branches, and isolated from surrounding neurons. *Z*-stack images were uploaded to the Reconstruct software (Version 1.1.0.0, 2007, The University of Texas at Austin).

For soma size analysis only, 40 single neuron images from the CA1 region of the hippocampus and layer II/III of the prefrontal cortex of both strains (10 neurons per mouse) were captured in an Olympus Bx43 microscope (Olympus, Center Valley, PA, United States; Objective Microscope: Olympus UMPlanFl 40 40 × 0.75 Infinity/0 UM Plan FL) coupled to a Micro-Publisher 5.0 RTV photographic camera (QImaging, Surrey, BC, Canada).

### Spine Density Analysis

For the spine density analysis, six 25-μm dendritic segments from the apical and basal dendrites of 20 neurons per strain (five neurons per mouse) were randomly selected. Three segments were from the proximal part of the apical dendrites (distance < 40 μm from the soma), and the three remaining segments were taken from the distal region (distance > 40 μm from the soma). Regarding the basal dendrite segments, the same selection was made in order to get a representative sample of the spines from the whole dendritic arbor of each neuron. We analyzed a total of 12 dendritic segments per neuron, and each spine was manually counted using the Reconstruct software.

### Soma Perimeter, Area, and Circularity Index Measurement

For both strains, 40 neuronal somas from each brain area were analyzed (40 neurons per strain, 10 neurons per mouse). Soma border was manually selected using the Reconstruct software, and perimeter and area were automatically calculated. The circularity index was also estimated by dividing the area by perimeter squared, and the result was multiplied by 4 times *pi*. A circularity index value of 1.0 indicates a perfect circle.

### Evaluation of Spine Length and Width

Ten dendritic segments (10 μm each) from the apical and basal dendrites of 20 neurons per strain (five neurons per mouse) were randomly selected for the spine morphology analysis using the Reconstruct software. Five segments from the proximal part and five segments from the distal portion of both apical and basal dendrites were studied. Thus, a total of 20 dendritic segments were analyzed per neuron. We collected data for two parameters of the spine shape: length and width. According to the *x-* and *y*-axis of the working field, only dendritic spines laterally extended from the selected dendrite segment were considered to avoid dimension distortion. The length was measured from the base of the spine to the tip. Defined as the broadest part of the spine, width was measured across the head. The morphology of approximately 6,000 spines per mouse strain was studied.

Histograms were made for the frequency distribution of all length and width spine measurements from both mouse strains. The number of histogram bins was determined by calculating the square root of the total number of observations (N) in the apical and basal dendrites (58 bins for the hippocampus and 50 bins for the prefrontal cortex).

Probability (relative frequency) was also estimated by dividing frequency values by the total number of observations (N) of each strain. Then, by adding all probability values, ogives of the cumulative probability distribution of spine length and width parameters were calculated.

### Classification of Spines by Shape Type

First, morphological evaluation of dendritic spines was based on previously reported data of spine size. We established two classification criteria based on the length of the *filopodia shape*, which is considered a long immature structure (Fiala et al., 1998, 2002; Yuste and Bonhoeffer, 2004; Gipson and Olive, 2017), and according to the head width of the *mushroom shape*, which is recognized as the most mature and functional spine morphology (Sorra and Harris, 2000; Bourne and Harris, 2007, 2008; Berry and Nedivi, 2017).

Thereby, using RStudio 1.3 (RStudio, Boston, MA, United States), dendritic spines were classified as filopodia-like (length > 2 μm) or non-filopodia-like spines (length ≤ 2 μm) according to their length, and as mushroom head-like (width > 0.6 μm) or non-mushroom head-like (width ≤ 0.6 μm) according to their width.

We performed a second analysis of spine morphology following the widely used protocol proposed by Risher et al. (2014), with some modifications to their classifying formulas to execute a more restricted evaluation. Spine shape was categorized according to their length, width, and length/width ratio (LWR) into seven types: filopodia (length > 2 μm; LWR > 1; width ≤ 0.6 μm), long thin (length ≤ 2 μm; length > 1 μm; width ≤ 0.6 μm), thin (length ≤ 1 μm; width ≤ 0.6 μm), stubby (LWR ≤ 1), mushroom with a medium head (width > 0.6 μm), mushroom with a wide head (width > 0.6 μm; LWR < 1), and branched spines (two heads attached to a single spine neck were tagged and counted as one “branched spine” with the Reconstruct software).

### Clustering of Spines by Length and Width Dimensions

Dendritic spines were also clustered to define the principal groups according to their own width and length features using RStudio 1.3. We used the K-means algorithm, in which the center of the established clusters (centroids) represents the group mean, and each data point is added to its closest k-centroid. Width and length data were scaled by subtracting the mean of the variable and dividing the result by the standard deviation of the variable. The number of optimal clusters (*k* = 3) was determined by the Within-Cluster-Sum of the Squared Errors (WSS) and Silhouette methods.

Dendritic spines from both brain areas of C58/J and neurotypical mice were grouped into three main clusters.

Clusters were defined as *small* (hippocampus: mean width = 0.42 μm, mean length = 0.501 μm; prefrontal cortex: mean width = 0.503 μm, mean length = 0.629 μm), *long* (hippocampus: mean width = 0.555 μm, mean length = 1.46 μm; prefrontal cortex: mean width = 0.644 μm, mean length = 1.69 μm), and *wide* (hippocampus: mean width = 0.789 μm, mean length = 0.893 μm; prefrontal cortex: mean width = 0.925 μm, mean length = 1.12 μm) spines, according to their length and width parameters. The distribution of dendritic spines among the three clusters was compared between C58/J and neurotypical strains.

### Evaluation of Hippocampal BDNF Content

BDNF (brain-derived neurotrophic factor) protein content was determined in the hippocampus of both mouse strains using the Western blot technique. The hippocampus of four mice per strain (bregma -2.03 to -2.27 mm) was homogenized in 500 μl of lysis buffer with protease inhibitors (10 mM Tris–HCl, 1 mM dithiothreitol, 30% glycerol, 1% Triton X-100, 15 mM sodium azide, 1 mM EDTA, 2 g/ml leupeptin, 2 g/ml aprotinin, 1 mM PMSF). Then, total protein samples were obtained by centrifugation of 15,000 rpm, 4°C for 15 min, and quantified with a Nanodrop 2000 (Thermo Fisher Scientific Inc., Wilmington, DE, United States). Proteins (100 μg) were separated by electrophoresis on 15% SDS-PAGE at 80 mV. Protein Standards (Bio-Rad, Hercules, CA, United States) were included for molecular weight determination. Gels were transferred to nitrocellulose membranes (Amersham; Sigma–Aldrich, St. Louis, MO, United States) in semi-dry conditions for 2 h at 60 mA. Membranes were blocked with 5% non-fat dry milk and 2% albumin for 2 h at room temperature and incubated with an antibody against BDNF (rabbit polyclonal anti-BDNF sc-546; Santa Cruz Biotechnologies, Santa Cruz, CA, United States; 0.34 μg/ml) for 24 h at 4°C. Afterward, the BDNF blot was incubated with goat anti-rabbit IgG conjugated to horseradish peroxidase [HRP] (111-035-003; Jackson Immuno Research, West Grove, PA, United States; dilution 1:15,000) for 45 min. Chemiluminescence signals were detected by exposing the membranes to Kodak Biomax Light Film (Sigma–Aldrich) using Supersignal West Femto as peroxidase substrate (Thermo Fisher Scientific, Waltham, MA, United States).

Next, BDNF blot was stripped with glycine (0.1 M, pH 2.5, 0.5% SDS) overnight at 4°C and re-probed for the α-tubulin loading control protein [mouse monoclonal anti-α-tubulin, sc-398103, Santa Cruz; 0.4 μg/mL; secondary antibody: goat anti-mouse IgG conjugated to horseradish peroxidase (HRP), AP127P, Sigma–Aldrich, MO, United States; dilution 1:15 000].

Semi-quantitative analysis of protein content was performed for the densitometry values of BDNF and α-tubulin blots using the ImageJ 1.45S software (National Institutes of Health, Bethesda, MD, United States).

### SNPs Retrieval and Gene Ontology (GO) Enrichment Analysis

We retrieved SNPs (single-nucleotide polymorphisms) information of C58/J and C57 BL/6J mice from The Mouse Phenome Database (MPD) (Bogue et al., 2020; The Jackson Laboratory, 2021). For our analysis, the UCLA1 data set developed by Eleazar Eskin at the University of California, which provides the SNP profiling of 132,285 genomic locations of 248 mouse strains (Eskin, 2018), was selected.

We scanned the entire genome of both strains specifying the preference for *Coding non-synonymous Cn* (resulting in changes in amino acid sequence) and *Coding synonymous Cs* (resulting in non-change of amino acid sequence) sequence variants. Data of polymorphisms in the C58/J strain genome in comparison to C57 BL/6J mice were downloaded. Single-nucleotide differences in 454 genes were detected in C58/J mice relative to the C57 BL/6J strain.

In order to identify the main processes in which the products of these genes were involved and the relationship between them, we performed a Gene Ontology (GO) enrichment analysis using the open-source GOnet web application developed by the Bjoern Peters Lab at La Jolla Institute for Allergy and Immunology, La Jolla, CA (available at https://tools.dice-database.org/GOnet/) (Pomaznoy et al., 2018). The 454 polymorphic genes were submitted to the GOnet platform (using Uniprot ID), and the GO term enrichment analyses for *biological process* and *molecular function* ontologies (*q*-value threshold: *p* < 0.05; background: all annotated genes) were executed.

For the *biological process* and *molecular function* categories, 319 and 43 GO terms were identified, respectively. We selected 23 GO *biological process* terms, and six GO *molecular function* terms associated with cytoskeleton organization, neuronal and synapse function, signaling, central nervous system development, and cognitive processes.

The polymorphic genes in the final selected GO terms were searched at the SFARI GENE database (Simons Foundation Autism Research Initiative, 2020) to detect if related orthologous human genes associated with autism spectrum disorder were found.

### Statistical Analysis

The results were plotted and analyzed using RStudio 1.3.

The comparison between the cumulative probability distributions of width and length was performed using the Kolmogorov-Smirnov test. The comparison of soma dimensions, spine density, shape classification, and cluster distribution between mouse strains was performed using an unpaired two-tailed Student’s *t*-test. Holm–Bonferroni was used for *p*-value correction. A *p* value under 0.05 was considered significant.

For GO enrichment analysis, GOnet establishes that for every GO term considered, the *p*-value in the Fisher exact test is computed. *Q*-value threshold: a *p* value under 0.05. All genes annotated were used as background.

## Results

To evaluate the neuronal morphology, we stained the whole soma and the dendritic tree of neurons from the CA1 region of the hippocampus and the II/III layer of the prefrontal cortex from both mouse strains using the Golgi-Cox staining protocol previously described.

**FIGURE 1:**
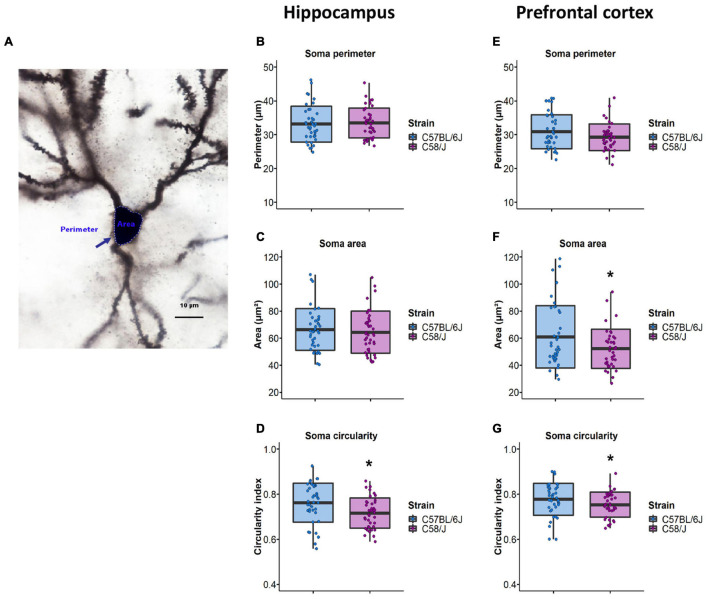
Neuronal soma size analysis in the hippocampus and the prefrontal cortex of C58/J mice. Representative image of soma perimeter and area **(A)**. Perimeter, area, and circularity index of pyramidal neurons soma in the hippocampus **(B–D)** and the prefrontal cortex **(E–G)** of C57 BL/6J (neurotypical phenotype) and C58/J (autistic-like phenotype) mice. *n* = 40 neurons per strain, 10 neurons per mouse. *t*-test: *p < 0.05.

First, we assessed the neuronal soma size by analyzing its perimeter and area (Figure 1A). There were no differences in soma size in the hippocampus between both strains (Figures 1B,C). However, the prefrontal cortex of the C58/J autistic-like mice showed a reduced soma area compared to the C57 BL/6J neurotypical group (Figures 1E,F). We also calculated the circularity index, an indicator of soma roundness. A lower circularity index was observed in both brain areas of C58/J strain compared to the neurotypical mice, which may suggest less rounded somas in the autistic-like mice (Figures 1D,G).

**FIGURE 2:**
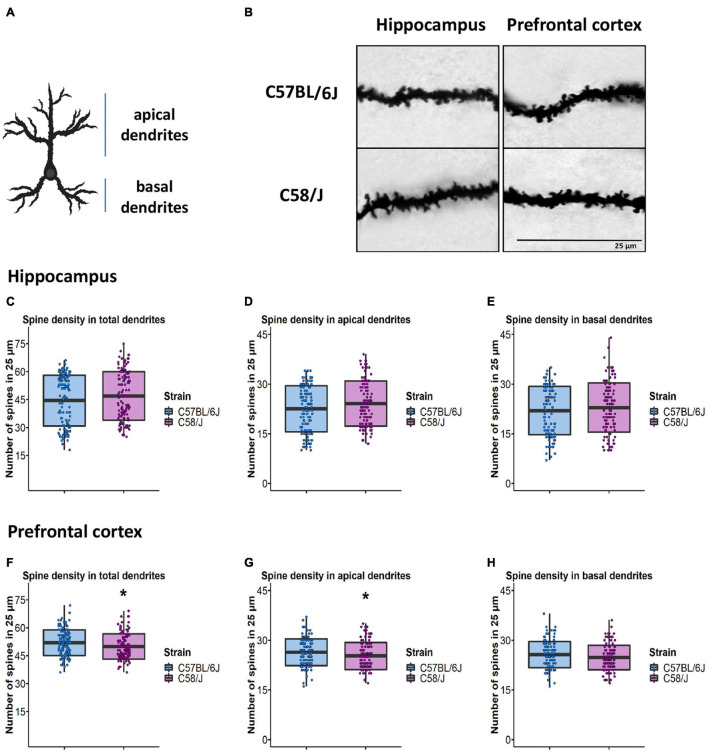
Determination of spine density in the pyramidal neurons of the hippocampus and the prefrontal cortex of C58/J mice. Scheme of apical and basal dendrite location **(A)**. A representative image of dendritic spines in the hippocampus and the prefrontal cortex of C57 BL/6J (neurotypical phenotype) and C58/J (autistic-like phenotype) mice **(B)**. Quantification of spine density per 25 μm in total, apical, and basal dendrites of the hippocampus **(C–E)** and the prefrontal cortex **(F-H)** in both mouse strains. Results are expressed as mean ± SD; *n* = 20 neurons per strain, five neurons per mouse. *t*-test: *p < 0.05.

Since dendrites of pyramidal neurons receive and process different stimuli depending on their location (Megías et al., 2001; Little and Carter, 2012), we decided to perform dendritic spine analysis by dividing neurons into two compartments: apical and basal dendrites (Figure 2A). Furthermore, we picked segments from the proximal and distal parts of the dendrites respecting soma to get a representative sample of spines throughout each neuron’s dendritic tree.

## Spine Density Determination

For spine density determination, we selected 25-μm dendritic segments from the apical and basal locations. All spines along the dendritic section were manually counted using the Reconstruct software for both brain areas (Figures 2A,B).

We did not observe differences in the number of dendritic spines per 25 μm in either the apical or basal dendrites of pyramidal neurons from the hippocampus of C58/J mice compared to the C57 BL/6J strain (Figures 2C–E). Interestingly, we detected a lower number of spines per 25 μm in the dendrites from the prefrontal cortex of the C58/J autistic-like mice in comparison with the neurotypical group; such difference was particularly displayed by apical dendrites. There were no significant changes in the basal dendrites of the prefrontal cortex between both strains (Figures 2F–H).

## Analysis of Spine Length and Head Width

We collected data on the total length of the spines and their head width to analyze the morphological features of the spines of each mouse strain.

First, we evaluated the distribution of the length and width data of the spines in both mice groups. Spine length in the apical and basal dendrites was in the range of 0–4 μm in the hippocampus and the prefrontal cortex of both strains. Additionally, the spine head width in the apical and basal dendrites was at an interval of 0–2 μm in the hippocampus and the prefrontal cortex of both mice groups (Supplementary Figures 1, 2).

**FIGURE 3:**
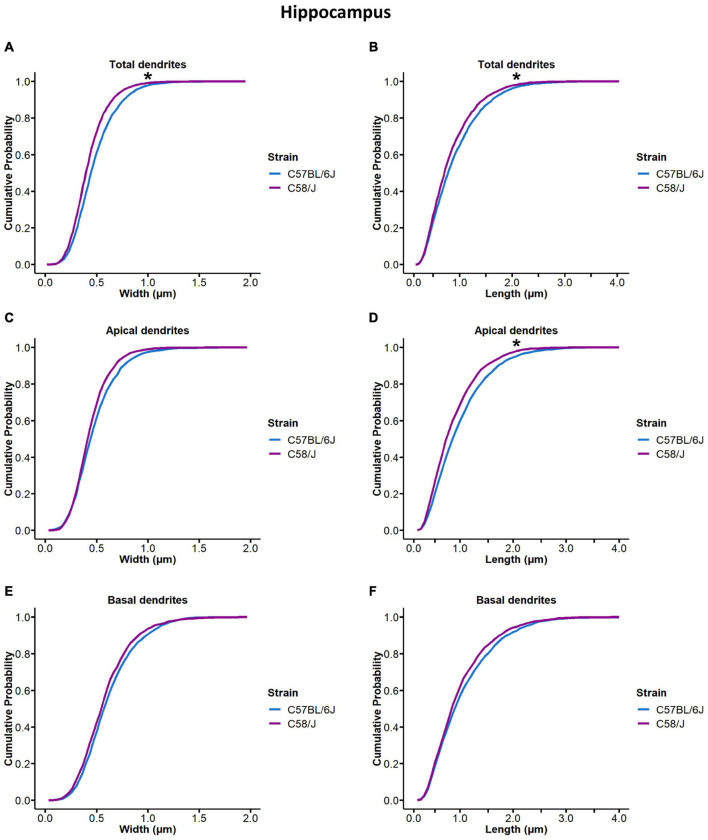
Cumulative probability distribution of spine width and length measurements in the hippocampus of C58/J mice. Cumulative probability distribution ogives of spine width and length measurements in total **(A,B)**, apical **(C,D)**, and basal **(E,F)** dendrites of pyramidal neurons in the hippocampus of C57 BL/6J (neurotypical phenotype) and C58/J (autistic-like phenotype) mice. *n* = 20 neurons per strain, five neurons per mouse. Kolmogorov–Smirnov test: *p < 0.05.

Cumulative probability distributions were compared to identify changes in length and width parameters between both strains (Figure 3). We observed different distributions of spine width and length in the hippocampus of the C58/J autistic-like mice in comparison with the C57 BL/6J neurotypical strain (Figures 3A,B). When dendrites were analyzed according to their location, significant differences in spine length were found in the apical dendrites (Figures 3C–F). However, cumulative probability distributions of spine dimensions were similar in the prefrontal cortex of both mice (Supplementary Figure 3).

## Classification of Dendritic Spines by Shape-Type

**FIGURE 4:**
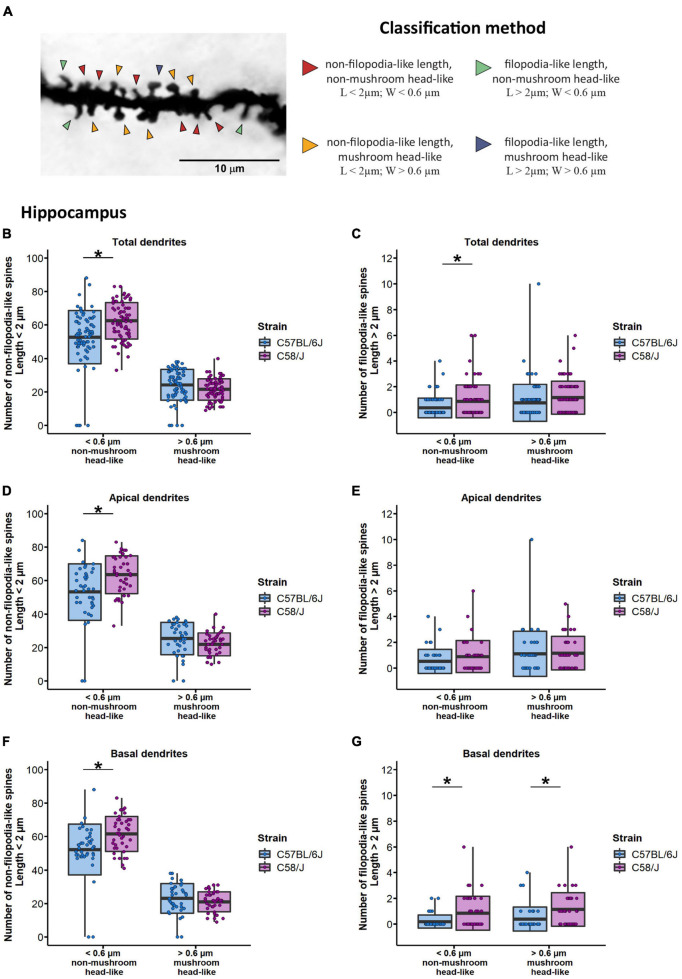
Classification of dendritic spines by shape type in the hippocampus of C58/J mice. Representative image of spine classification by shape type **(A)**. Arrowheads are pointing to examples of each shape type. Dendritic spines were categorized depending on their length into filopodia-like or non-filopodia-like, and by their width, as mushroom head-like or non-mushroom head-like, in total **(B,C)**, apical **(D,E)**, and basal **(F,G)** dendrites of pyramidal neurons in the hippocampus of C57 BL/6J (neurotypical phenotype) and C58/J (autistic-like phenotype) mice. Results are expressed as mean ± SD; *n* = 20 neurons per strain, five neurons per mouse. *t*-test with Holm–Bonferroni correction: *p < 0.05.

Once spinal width and length distribution changes were identified, we aimed to analyze more specific alterations on spine shape based on mature (mushroom) and immature (filopodia) morphology features. Dendritic spines were categorized depending on their length into filopodia-like (length > 2 μm) or non-filopodia-like (length ≤ 2 μm) spines, and by their width, as mushroom head-like (width > 0.6 μm) or non-mushroom head-like (width ≤ 0.6 μm), according to sizes previously reported (Fiala et al., 1998, 2002; Sorra and Harris, 2000; Bourne and Harris, 2007, 2008; Gipson and Olive, 2017; Kaul et al., 2020; Figure 4A). We observed a higher number of non-mushroom head-like spines with either non-filopodia-like or filopodia-like length in C58/J mice dendrites compared to the neurotypical strain (Figures 4B,C). When dendrites were analyzed according to their location, non-mushroom head-like spines with non-filopodia-like length were increased in both apical and basal dendrites of the autistic-like mice (Figures 4D,F). The number of filopodia-like length spines with any head size was also increased in the basal dendrites of the C58/J mice compared to the C57 BL/6J neurotypical group (Figure 4G). No change was observed in the number of filopodia-like length spines from the apical dendrites (Figure 4E). Furthermore, there were no significant differences in the shape of spines from the prefrontal cortex between both strains (Supplementary Figure 4).

Moreover, the classification protocol proposed by Risher and collaborators is one of the two-dimensional (2-D) methods that have been widely used to study the morphology of dendritic spines. This analysis was standardized in Golgi–Cox-stained tissue from the mouse brain, and it is based on distinctive geometric characteristics of the spine (Risher et al., 2014).

For our study, we also performed Risher’s classification method with some modifications (detailed in section “Materials and Methods”). Dendritic spines were grouped into seven shape categories: filopodia, long-thin, thin, stubby, mushroom with a medium head, mushroom with a wide head, and branched spines, according to their length, width, and length/width ratio (Supplementary Figure 5a). We found an increased number of filopodia, long-thin, and thin spines in the hippocampal dendrites of the C58/J autistic-like mice compared to the neurotypical group (Supplementary Figure 5b). After analyzing dendrites according to their location, a higher number of long-thin spines was specifically observed in the hippocampal basal dendrites of autistic-like mice (Supplementary Figures 5c,d). We did not observe any significant change in the spine morphology between the prefrontal cortex of both strains (Supplementary Figures 5e–g).

## Clustering of Spines by Length and Width Dimensions

Next, we explored the morphology of dendritic spines by grouping them according to their own width and length features using the *K*-means clustering approach.

**FIGURE 5:**
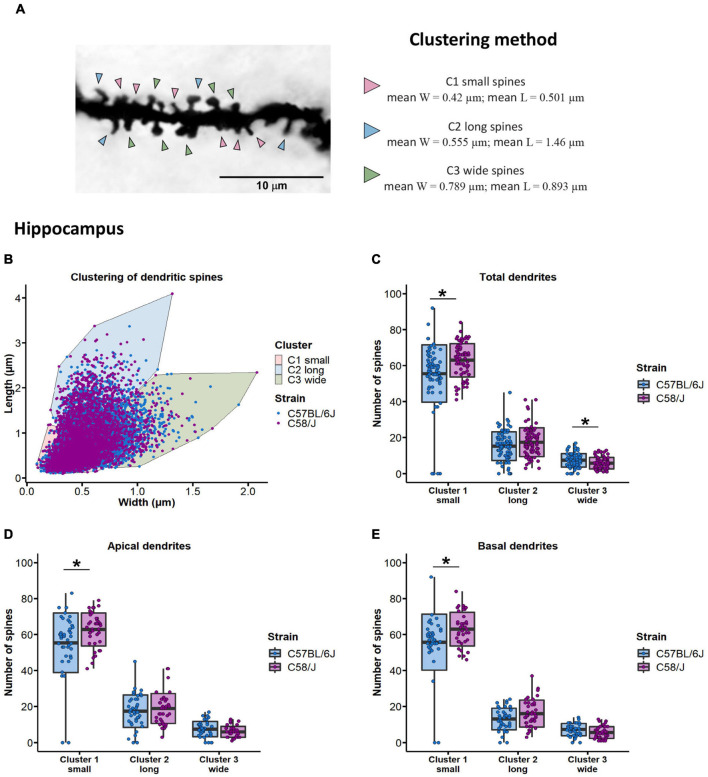
Clustering of dendritic spines by length and width dimensions in the hippocampus of C58/J mice. Dendritic spines are grouped in three clusters **(A)**. Arrowheads are pointing to examples of *small*, *long*, and *wide* spines. Distribution of dendritic spines in three main clusters using the K-means algorithm **(B)**. Clusters were defined as *small*, *long*, and *wide* spines, according to their length and width parameters. Number of dendritic spines grouped in each cluster, in total **(C)**, apical **(D)**, and basal **(E)** dendrites of pyramidal neurons in the hippocampus of C57 BL/6J (neurotypical phenotype) and C58/J (autistic-like phenotype) mice. Results are expressed as mean ± SD; *n* = 20 neurons per strain, five neurons per mouse. *t*-test with Holm–Bonferroni correction: *p < 0.05.

Three optimal clusters were established for dendritic spines from the hippocampus and the prefrontal cortex of both mouse strains. Depending on the width and length parameters of each cluster, we defined the shape of dendritic spines as *small* if they were grouped in cluster 1 (hippocampus: mean width = 0.42 μm, mean length = 0.501 μm; prefrontal cortex: mean width = 0.503 μm, mean length = 0.629 μm), *long* spines if included in cluster 2 (hippocampus: mean width = 0.555 μm, mean length = 1.46 μm; prefrontal cortex: mean width = 0.644 μm, mean length = 1.69 μm), and *wide* spines if they were included in cluster 3 (hippocampus: mean width = 0.789 μm, mean length = 0.893 μm; prefrontal cortex: mean width = 0.925 μm, mean length = 1.12 μm) (Figures 5A,B and Supplementary Figure 6).

We observed an increased number of small spines and a lower number of wide spines in the hippocampal dendrites of C58/J autistic-like mice in comparison with the C57 BL/6J neurotypical strain (Figure 5C). When dendrites were divided according to their location, a higher number of small spines was observed in both apical and basal dendrites of autistic-like mice, without significant changes in the number of long and wide spines compared to the neurotypical group (Figures 5D,E). No changes were observed in the distribution of the dendritic spines among clusters in the prefrontal cortex of both mouse groups (Supplementary Figure 6).

## Evaluation of Hippocampal BDNF Content

**FIGURE 6:**
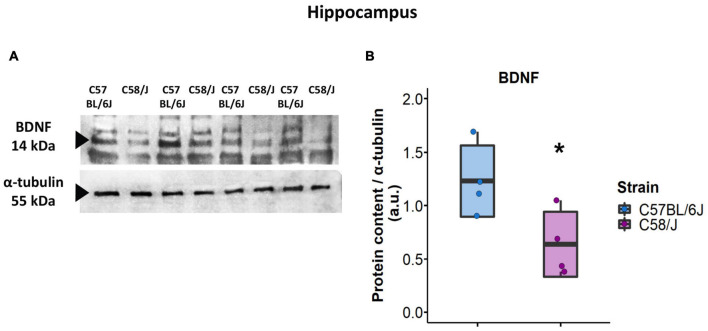
BDNF protein content in the hippocampus of C58/J mice. Western blot of BDNF content in the hippocampus of C57 BL/6J (neurotypical phenotype) and C58/J (autistic-like phenotype) mice **(A)**. Semi-quantitative analysis of BDNF densitometry for both strains **(B)**. Results are expressed as mean ± SD; *n* = 4 mice per strain. *t*-test: *p < 0.05.

Since the hippocampus of C58/J mice showed the most significant modifications in dendritic spine morphology, we also evaluated the protein content of BDNF (brain-derived neurotrophic factor) in the total lysate of this brain area by a semi-quantitative Western blot analysis. The analysis showed a lower expression of BDNF protein in the hippocampus of autistic-like mice compared to the C57 BL/6J neurotypical group (Figure 6).

## *In Silico* Identification of Single-Nucleotide Polymorphisms in the C58/J Mice Genome

We searched for SNPs in the genome of the C58/J autistic-like strain in comparison with the C57 BL/6J neurotypical mice. The polymorphism data were retrieved from The Mouse Phenome Database (MPD). SNPs in 454 genes were identified when comparing both mouse strains (Supplementary Table 1). We submitted these genes to the GOnet platform for a GO enrichment analysis for *biological process* and *molecular function* ontologies.

Polymorphic genes between C58/J and C57 BL6/J strains were detected as part of 319 GO terms from the *biological process* category, and 43 terms from the *molecular function* category (Supplementary Table 2).

We selected 23 GO *biological process* terms and 6 GO *molecular function* terms associated with cytoskeleton organization, neuronal and synapse function, signaling, central nervous system development, and cognitive processes. Forty-five polymorphic genes shared at least five *biological process* and/or *molecular function* selected terms (Supplementary Table 3). Moreover, the C58/J mice polymorphic genes were searched at the SFARI database, and we found that 11 of them (NF1, SLC6A4, MAP1A, NTRK2, MYO9B, CNTNAP2, PLCB1, VIL1, ATP2B2, LRP1, ESR2) had orthologous human genes associated with ASD risk (Table 1). Additionally, we also identified seven genes involved in four or less selected GO terms with an ASD risk-related orthologous human gene (CIC, GRM7, STK39, DHCR7, ANKRD11, LEO1, CPZ) (Table 1).

**TABLE 1:** GO enrichment analysis of C58/J mice polymorphic genes with orthologous human genes associated with ASD-risk.

GO Biological process/Molecular function	Uniprot ID	Gene symbol	Gene name	Orthologous human gene in SFARI	Uniprot ID	SFARI score
1, 2, 3, 7, 12, 13, 14, 15, 16, 18, 19, 20, 21, 23	Q04690	NF1	Neurofibromin 1	NF1	P21359	1
6, 7, 12, 13, 14, 18, 19, 1^*^, 2^*^, 3^*^, 4^*^, 5^*^	Q60857	SLC6A4	Solute carrier family 6 (neurotransmitter transporter, serotonin), member 4	SLC6A4	P31645	3
1, 4, 5, 9, 10, 11, 12, 18, 19, 1^*^, 2^*^	Q9QYR6	MAP1A	Microtubule-associated protein 1A	MAP1A	P78559	1
8, 11, 12, 13, 14, 15, 16, 18, 19, 20, 21	P15209	NTRK2	Neurotrophic tyrosine kinase, receptor, type 2	NTRK2	Q16620	S
3, 20, 21, 23, 1^*^, 2^*^, 3^*^, 4^*^, 5^*^, 6^*^	Q9QY06	MYO9B	Myosin IXb	MYO9B	Q13459	2
9, 11, 12, 13, 14, 15, 18, 19	Q9CPW0	CNTNAP2	Contactin associated protein-like 2	CNTNAP2	Q9UHC6	2S
13, 14, 15, 16, 18, 19, 20, 21	Q9Z1B3	PLCB1	Phospholipase C, beta 1	PLCB1	Q9NQ66	2
1, 2, 3, 21, 1^*^, 2^*^, 3^*^	Q62468	VIL1	Villin 1	VIL1	P09327	2
8, 9, 11, 12, 13, 14, 19	Q9R0K7	ATP2B2	ATPase, Ca++ transporting, plasma membrane 2	ATP2B2	Q01814	2
9, 10, 11, 12, 13	Q91ZX7	LRP1	Low density lipoprotein receptor-related protein 1	LRP1	Q07954	2
12, 13, 14, 18, 19	O08537	ESR2	Estrogen receptor 2 (beta)	ESR2	Q92731	3
13, 14, 18, 19	Q924A2	CIC	Capicua transcriptional repressor	CIC	Q96RK0	1
18, 19, 21	Q68ED2	GRM7	Glutamate receptor, metabotropic 7	GRM7	Q14831	3
20, 21	Q9Z1W9	STK39	Serine/threonine kinase 39	STK39	Q9UEW8	3
17	O88455	DHCR7	7-dehydrocholesterol reductase	DHCR7	Q9UBM7	1
17	E9Q4F7	ANKRD11	Ankyrin repeat domain 11	ANKRD11	Q6UB99	1
21	Q5XJE5	LEO1	Leo1, Paf1/RNA polymerase II complex component	LEO1	Q8WVC0	2
21	Q8R4V4	CPZ	Carboxypeptidase Z	CPZ	Q66K79	3

*Biological process. 1: cytoskeleton organization, 2: actin cytoskeleton organization, 3: actin filament-based process, 4: microtubule-based process, 5: microtubule-based movement, 6: neurotransmitter reuptake, 7:neurotransmitter transport, 8: synapse organization, 9: neuron projection development, 10: axonogenesis, 11: neuron differentiation, 12: neurogenesis, 13: central nervous system development, 14: brain development, 15: forebrain development, 16: cerebral cortex development, 17: developmental growth, 18: learning or memory, 19: behavior, 20: intracellular signal transduction, 21: cell surface receptor signaling pathway, 22: integrin-mediated signaling pathway, 23: regulation of small GTPase mediated signal transduction.*

*Molecular function. 1^∗^: cytoskeletal protein binding, 2^∗^: actin binding, 3^∗^: actin filament binding, 4^∗^: small GTPase binding, 5^∗^: Ras GTPase binding, 6^∗^: Rho GTPase binding.*

## Discussion

Neurodevelopmental disorders, including autism spectrum disorder, have been widely involved with neurite anomalies (Penzes et al., 2011); therefore, we wanted to explore, in detail, the characteristics of dendritic spines in the C58/J inbred-mouse model of autism. Thus, we observed ASD-related structural changes in the whole neuronal structure that appear to be differentially expressed among brain areas and depending on the dendrite location in the C58/J autistic-like mice, including differences in soma shape, spine density, and morphology.

Before analyzing dendritic spines, we decided to study the changes in the neuronal soma dimensions between the C57 BL/6J neurotypical mice and the C58/J autistic-like strain. We found a reduced soma area only in the prefrontal cortex of the autistic-like strain. However, a lower circularity index was observed in the hippocampus and prefrontal cortex of the C58/J mice, which may implicate soma shape changes. Although we did not observe significant differences in each parameter in both brain regions of the autistic-like mice, the relationship between area and perimeter reflected in the circularity index suggested less rounded somas in C58/J mice. Accordingly, the analysis of post-mortem samples from people with an autism diagnosis has revealed small cell size in the entorhinal cortex, the hippocampus, and the amygdala (Bauman and Kemper, 2005; Wegiel et al., 2014).

Since approximately 90 percent of excitatory synapses occur in dendritic spines, their analysis could reflect a connectivity state of a specific brain area. Thus, changes in the number and structure of spines spread throughout the dendritic tree potentially affect synaptic transmission regulation and neuronal structural plasticity in general (Lefebvre et al., 2015).

We did not find spine density differences in the pyramidal neurons in the hippocampus of the C58/J autistic-like mice. Interestingly, in the prefrontal cortex, we observed a subtle reduction in the number of spines. It is known that when spine number variations are related to neural disorders, they could be associated with an impaired synaptic pruning process or the loss/gain of axonal synaptic contacts (Fiala et al., 2002). Since our density spine analysis was 2-D performed, further stereological assessments should be considered to estimate the total number of dendritic spines in each brain area.

The relationship between the morphology and functionality of spines is still being elucidated, but the dependence of synapse efficiency on the spine geometry characteristics has been proposed (Kasai et al., 2003). Thereby, we collected dendritic spine length and head width measurements from C58/J and C57 BL/6J strains. Changes in cumulative probability distributions were observed for spine length and width in the hippocampal dendrites of C58/J mice, suggesting spine morphology alterations.

First, we decided to use classification criteria based on the length of filopodia spines, considering a pioneer long immature structure that can give rise to mature spines (Yuste and Bonhoeffer, 2004; Gipson and Olive, 2017), and according to the head width of mushroom spines, which are the most mature and functional spine morphology since their head size often correlates with a prominent PSD with a higher number of post-synaptic receptors and synaptic proteins (Nimchinsky et al., 2002; Arellano, 2007; Bourne and Harris, 2007). Classification of dendritic spines by shape is often variable across studies and can also include specific morphologies such as mushroom, filopodia, thin, and stubby spines, such as in Risher’s classification method (Risher et al., 2014; Maiti et al., 2015).

In our study, the results of both classification methods revealed a higher frequency of longer immature spines in the hippocampus of C58/J autistic-like mice, either identified as filopodia-like or long-thin, along with more average-length spines without a mushroom-head, suggesting a lack of stable-mature spines in comparison with the neurotypical mice.

In addition to classification analyses, we clustered dendritic spines depending on their length and width dimensions to find the main groups in which spines shared morphological features. We observed that dendritic spines were distributed in three clusters—small, long, and wide spines. In the hippocampus of C58/J mice, a higher proportion of dendritic spines was grouped in the small-morphology cluster; by contrast, there was a reduced number of spines grouped in the wide-morphology cluster. Thereby, clustering of dendritic spines also showed an increased frequency of less mature-like shapes and a reduced number of spines with large mature-like heads, which might be associated with differences in the synaptic transmission efficacy in the hippocampus of autistic-like mice.

Furthermore, it must be mentioned that the decreased spine density observed in the prefrontal cortex of C58/J autistic-like mice cannot be attributed to the missing counting of dendritic spines due to a smaller size since we did not find changes in spine morphology.

There is evidence of dendritic spine alterations in other mouse models associated with ASD. For instance, in a valproic acid-induced mouse model of autism, an increased number of dendritic spines were found in primary cultures of cortical neurons (Yang et al., 2016). The MECP2 (methyl-CpG binding protein 2) duplication-mouse model (MECP2^Tg1^) has a higher spine density, along with a lower number of mushroom spines in the somatosensory and primary visual cortex (Wang et al., 2017). By contrast, the mouse model of 15q11-13-duplication (patDp/+) showed a reduced spine density, an increased number of thin spines, and a lower number of mushroom-shaped spines in the somatosensory cortex (Wang et al., 2017). Moreover, in the hippocampal neurons from mice with haploinsufficiency of the CYFIP1 gene (cytoplasmic FMR1 interacting protein 1), the number of immature-shaped spines was higher compared to the control group (Pathania et al., 2014). The CNTNAP2 (contactin-associated protein-like 2)- and DLGAP2 (DLG associated protein 2)-deficient mice also displayed fewer dendritic spines in the somatosensory and orbitofrontal cortex, respectively (Jiang-Xie et al., 2014; Gdalyahu et al., 2015). Similarly, the hippocampal pyramidal neurons of SHANK1 (SH3 and multiple ankyrin repeat domains 1) knockout mice show reduced spine density and size (Hung et al., 2008). Finally, it was proposed that the Fragile-X mouse model has an impaired immature to mature-spine turnover in the somatosensory cortex during postnatal development (Cruz-Martín et al., 2010).

In short, changes in dendritic spines are diverse across the models of autism, including ours. Nonetheless, it also seems to be one of the most recurrent anomalies that characterize syndromic and idiopathic ASD. Hence, it is essential to keep focusing on the study of the alterations of neuronal morphology and the molecular mechanisms that could be involved in the ASD-associated atypical neuronal plasticity.

Additionally, to explore a possible associated mechanism for morphology changes of dendritic spines in the hippocampus of C58/J mice, we analyzed the protein content of BDNF (brain-derived neurotrophic factor). Endogenous BDNF in hippocampal pyramidal neurons has been involved in the maintenance of the mature morphology of dendritic spines, and its absence is associated with an increased length of dendritic spines and a higher proportion of spines with a small head (Kellner et al., 2014; von Bohlen Und Halbach and von Bohlen Und Halbach, 2018). Accordingly, we observed a reduced content of BDNF in the hippocampus of autistic-like mice compared to the neurotypical group, which potentially contributes to the prevalence of immature-shaped dendritic spines. However, BDNF expression in each hippocampal region of C58/J mice must be further evaluated to identify differences in the content of this neurotrophin in the CA1 field compared to the others.

In order to find other related potential causes of dendritic spines changes in C58/J mice, we performed an *in silico* evaluation of polymorphisms in the genome of the autistic-like mice.

The Mouse Phenome Database (MPD) provides a comprehensive collection of phenotypic and genotypic data from inbred, recombinant inbred, F1 hybrid, and transgenic strains, among others (Bogue et al., 2020). Furthermore, SNP profiling for our experimental mouse groups is available in the MPD genotype dataset. Thus, the C58/J mice genome was scanned for polymorphisms in comparison with the C57 BL/6J strain, and we found a total of 454 genes with sequence differences between both mouse strains. It has been suggested that common genetic variants, including SNPs, contribute substantially to ASD susceptibility (Gaugler et al., 2014; Grove et al., 2019). Therefore, genetic variants found in C58/J mice might participate in their ASD-related neuronal alterations.

In accordance with the changes in spine density and morphology that we previously detected in the C58/J mice relative to the C57 BL/6J strain, we found polymorphic differences between both phenotypes in genes associated with cytoskeleton rearrangement and neurite development. Cytoskeleton dynamic modulation by several regulator proteins is essential for the formation and elongation of dendritic spines, synapse stabilization, and structural plasticity (Luo, 2002). Hence, variants at genes active in these processes might point to the genetic background of the C58/J strain associated with the observed dendritic spine anomalies.

Furthermore, we also observed SNPs at genes associated with central nervous system development, neurogenesis, and neuronal differentiation, along with ontologies likely involved in synapse function, such as neurotransmitter reuptake and signaling transduction. Polymorphisms in genes associated with behavior, learning, and memory were also observed.

Interestingly, the search for polymorphic genes of C58/J mice in the SFARI database showed 18 orthologous human genes associated with ASD risk. Both rare variants and SNPs have been reported in these genes in people with autism. Among these genes, we identified some with synaptic functions: MAP1A has a role in the development of dendritic spines and structural plasticity maintenance (Gu and Zheng, 2009); GRM7 is involved in mature glutamatergic synapses regulation (Noroozi et al., 2016); ANKRD11 participates in synapse signaling (Ka and Kim, 2018); SLC6A4 is implicated in serotoninergic transmission (Sener et al., 2019); and some others with less characterized neuronal roles, such as VIL1, ATP2B2, CIC, STK39, DHCR7, LEO1, and CPZ.

Thus, polymorphic genes of C58/J mice, in comparison with the genetic background of C57 BL/6J, could support the relationship between the genome of the C58/J strain, its autistic-like behavior, and the observed dendritic spines anomalies.

Previously, Moy et al. (2014) conducted an SNP analysis for the C58/J strain using the Center for Genome Dynamics (CGD) Strain Comparison Tool. They examined the distribution of 667 ASD-associated genes in C58/J mice at divergent and identical genomic regions in comparison with C57 BL6/J and C57 L/J strains. They found that 97 of these genes resided within genetic intervals unique to C58/J mice, including CNTNAP2, DISC1, SLC6A4, CACNA1C, GABRA5, and GABRB1. It must be mentioned that differences between Moy’s analysis and ours could be due to the different databases and the comparison of the third strain (C57 L/J). Thus, we decided to corroborate at least the SNPs of the 52 genes of our list, using the Sanger4 database (Wellcome Trust Sanger Institute., 2017), also available at the platform, and all of them were confirmed (Supplementary Table 4).

Additionally, the Mouse Phenome Database tools offer functional annotations for SNP location, such as *coding non-synonymous* and *coding synonymous* SNPs, along with others, using a variation effect prediction algorithm to determine the effect of nucleotide substitution in these genes on their encoded protein function. However, further experimental verification and characterization of these SNPs in the C58/J mice is required to analyze if protein function is compromised; if so, then the molecular mechanisms involved and their relationship with autistic phenotype should be explored.

In conclusion, C58/J mice display changes in dendritic spines, altered BDNF content, and SNPs at genes collectively involved in the regulation of structural plasticity with a potential association with ASD. Nonetheless, additional research of genetic, molecular, and behavioral features is guaranteed to understand their contribution to the idiopathic autistic-like phenotype of the C58/J strain.

## Data Availability Statement

The datasets presented in this study can be found in online repositories. The names of the repository/repositories and accession number(s) can be found in the article/Supplementary Material.

## Ethics Statement

The animal study was reviewed and approved by the local Institutional Animal Care and Research Advisory Committee (CICUAL, ID 189), from the Universidad Nacional Autónoma de México (UNAM), and performed under the International Guidelines of ethical care and use of animals, and the fulfillment of the Mexican procedure NOM 062 Z00.

## Author Contributions

IB-M and AG-A designed experiments, analyzed data, and wrote and reviewed the manuscript. IB-M performed all experiments. EM-M contributed to spinal density and western blot analyses. MD and MG-C contributed to samples’ preparation and processing. MM-M contributed to animal breeding, care, and management. All authors contributed to the article and approved the submitted version.

## Conflict of Interest

The authors declare that the research was conducted in the absence of any commercial or financial relationships that could be construed as a potential conflict of interest.

## Publisher’s Note

All claims expressed in this article are solely those of the authors and do not necessarily represent those of their affiliated organizations, or those of the publisher, the editors and the reviewers. Any product that may be evaluated in this article, or claim that may be made by its manufacturer, is not guaranteed or endorsed by the publisher.
