# Supplementary figures and images for: Changes in the Number and Morphology of Dendritic Spines in the Hippocampus and Prefrontal Cortex of the C58/J Mouse Model of Autism
Source: Front Cell Neurosci. 2021 Sep 20;15:726501. doi: 10.3389/fncel.2021.726501 (PMC8488392; doi:10.3389/fncel.2021.726501)

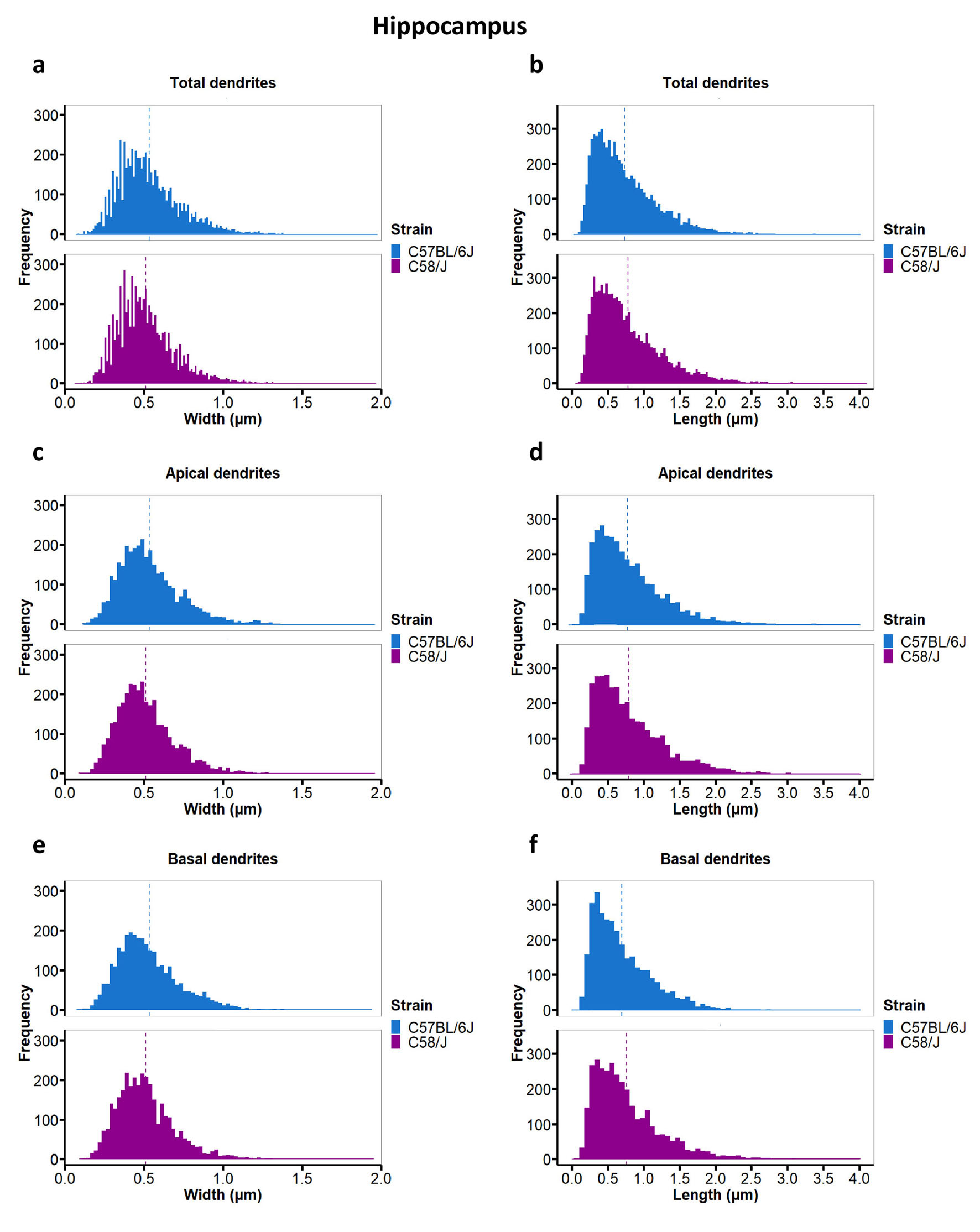

Supplement: Supplementary file 1 [file Image_1.JPEG]

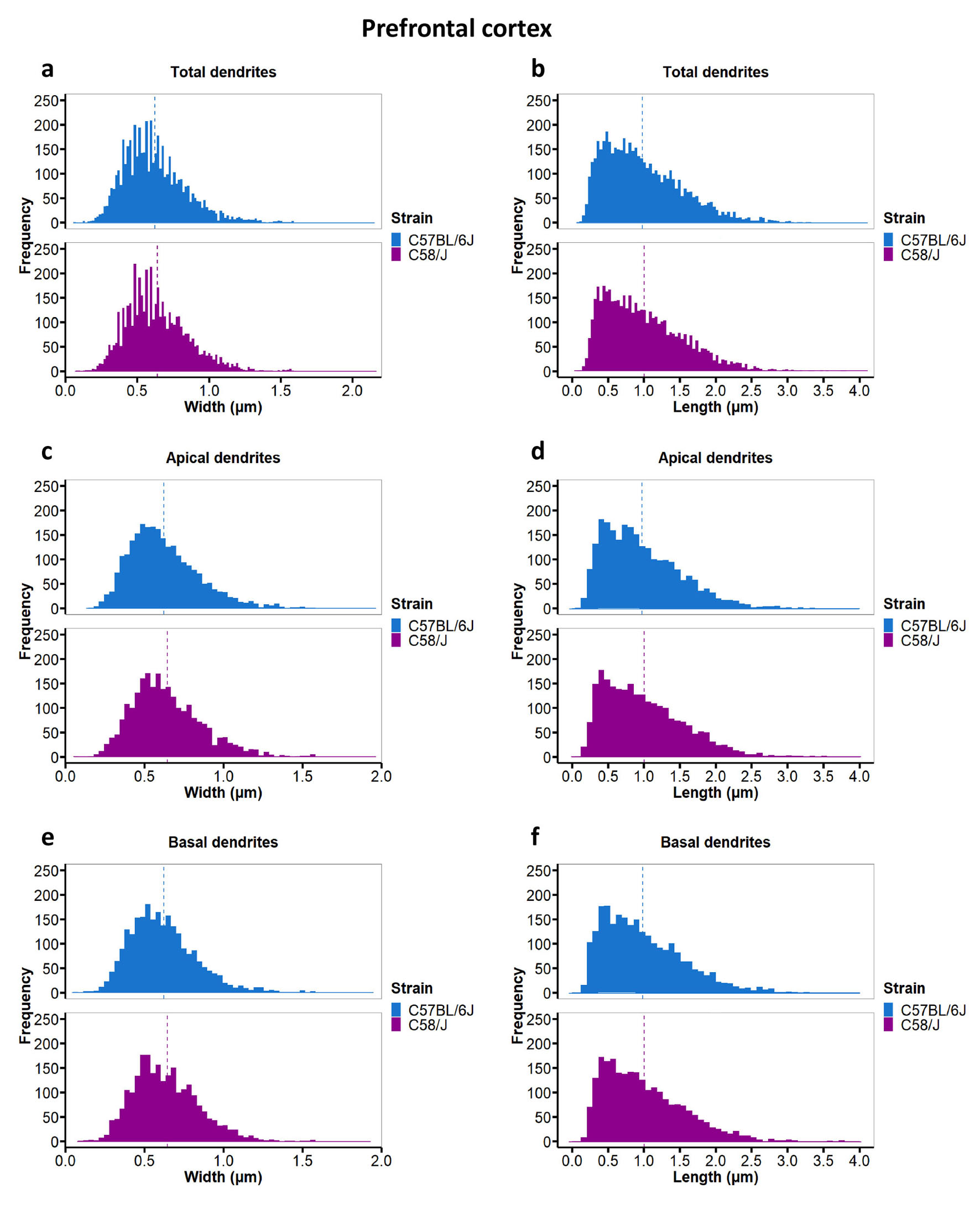

Supplement: Supplementary file 2 [file Image_2.JPEG]

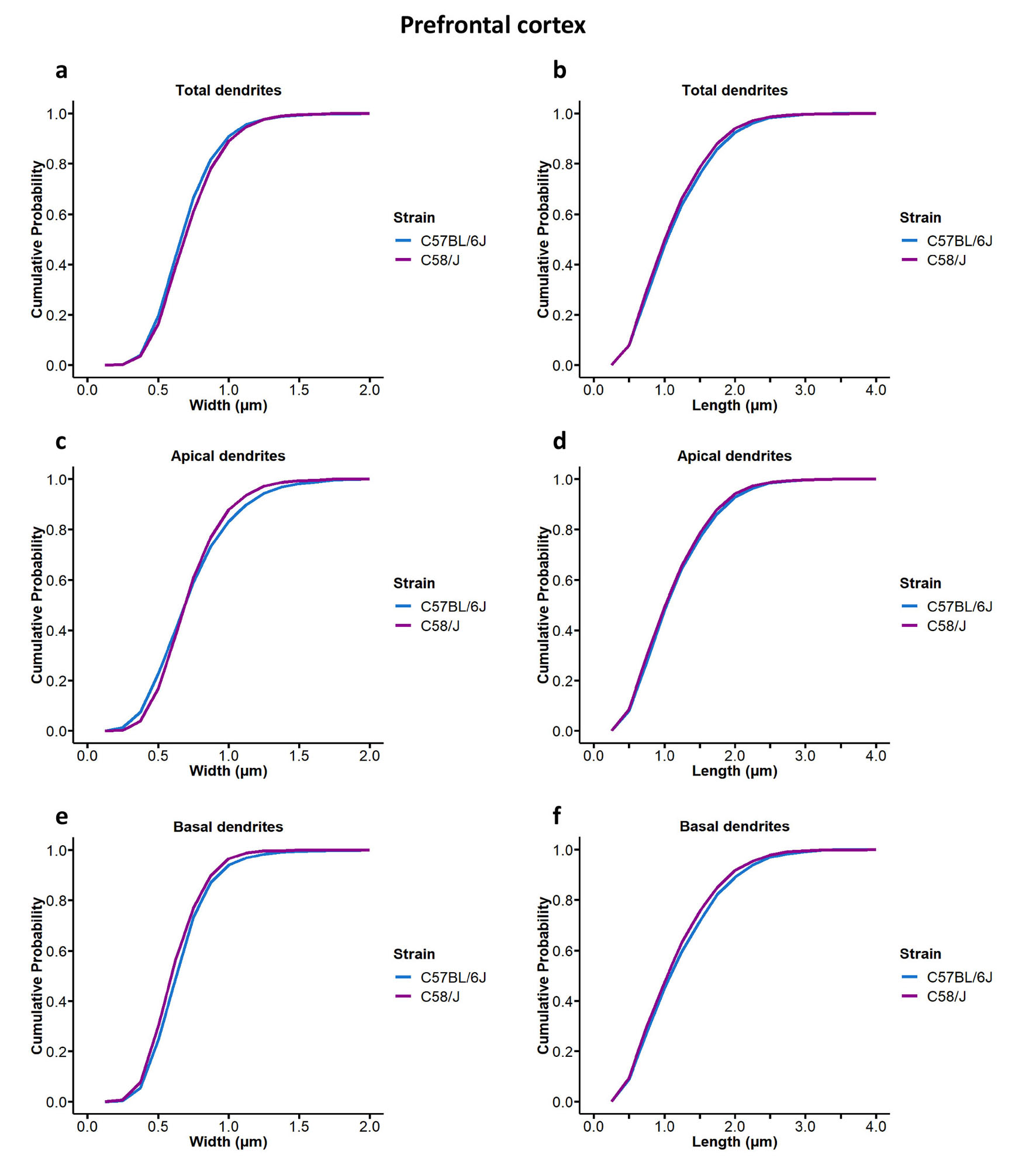

Supplement: Supplementary file 3 [file Image_3.JPEG]

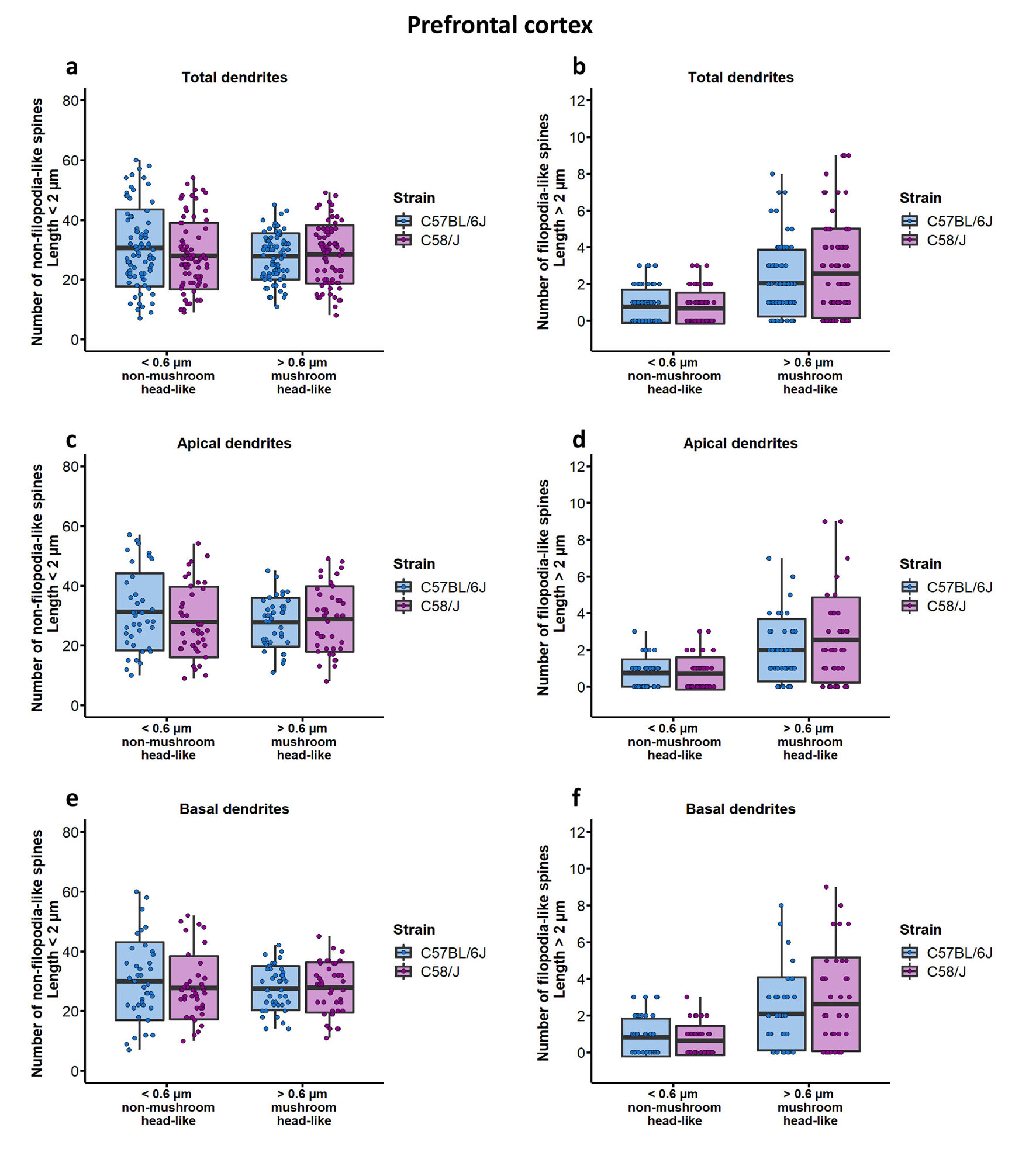

Supplement: Supplementary file 4 [file Image_4.JPEG]

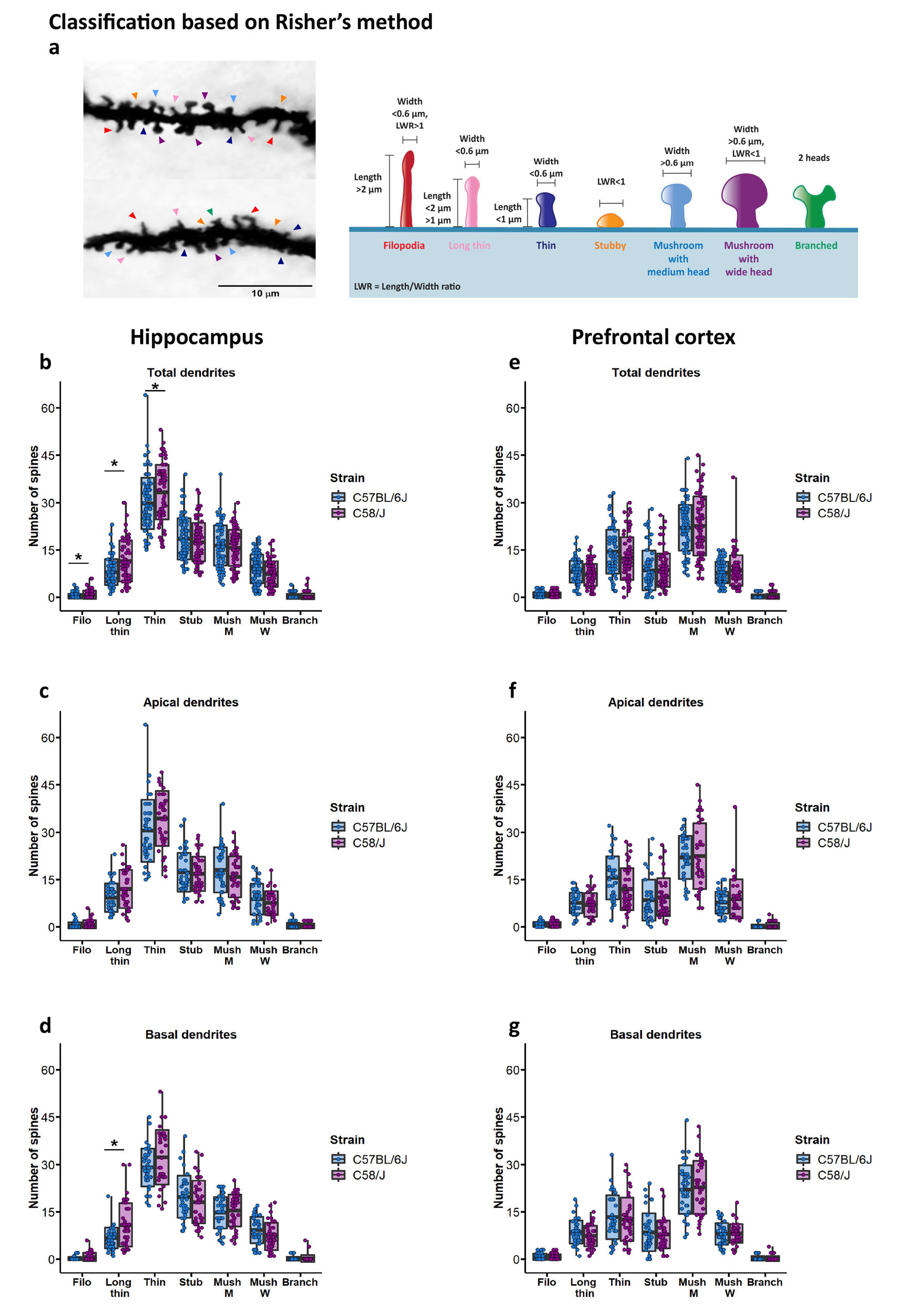

Supplement: Supplementary file 5 [file Image_5.JPEG]

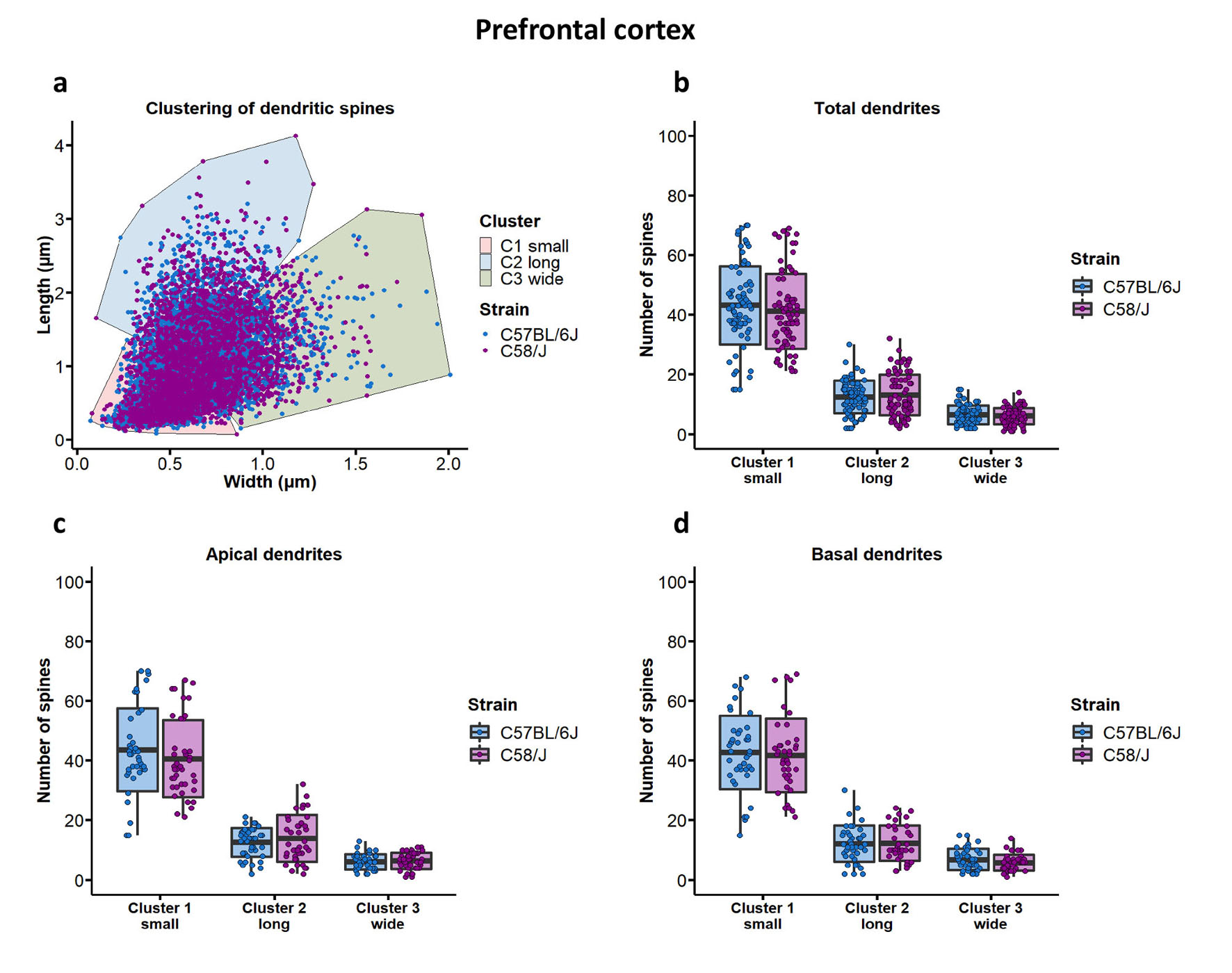

Supplement: Supplementary file 6 [file Image_6.JPEG]

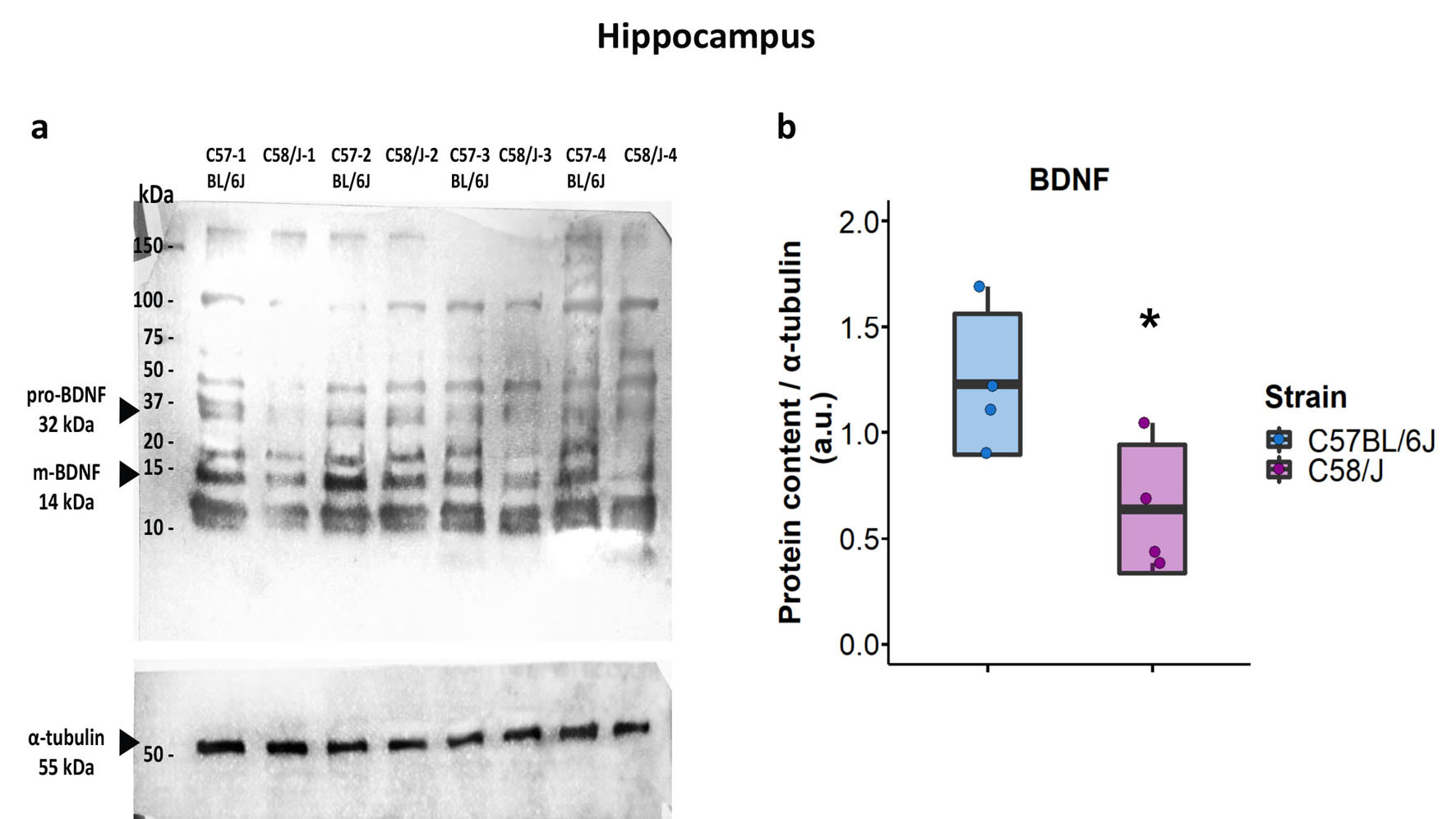

Supplement: Supplementary file 7 [file Image_7.JPEG]
