# Supplementary material for: Changes in the Number and Morphology of Dendritic Spines in the Hippocampus and Prefrontal Cortex of the C58/J Mouse Model of Autism
Source: Front Cell Neurosci. 2021 Sep 20;15:726501. doi: 10.3389/fncel.2021.726501 (PMC8488392; doi:10.3389/fncel.2021.726501)
Supplement: Supplementary file 8 [file Data_Sheet_1.zip › New folder (2)/Captions.DOCX]

**Supplementary Figures.**

**Supplementary Figure 1.**

**Distribution of spine width and length measurements in the hippocampus of C58/J mice.** Frequency distribution histograms of spine width and length measurements of total (a,b), apical (c,d), and basal (e,f) dendrites of pyramidal neurons in the hippocampus of C57 BL/6J (neurotypical phenotype) and C58/J (autistic-like phenotype) mice. n= 20 neurons per strain, 5 neurons per mouse.

**Supplementary Figure 2.**

**Distribution of spine width and length measurements in the prefrontal cortex of C58/J mice.** Frequency distribution histograms of spine width and length measurements of total (a,b), apical (c,d), and basal (e,f) dendrites of pyramidal neurons in the prefrontal cortex of C57 BL/6J (neurotypical phenotype) and C58/J (autistic-like phenotype) mice. n= 20 neurons per strain, 5 neurons per mouse.

**Supplementary Figure 3.**

**Cumulative probability distribution of spine width and length measurements in the prefrontal cortex of C58/J mice.** Cumulative probability distribution ogives of spine width and length measurements in total (a,b), apical (c,d), and basal (e,f) dendrites of pyramidal neurons in the prefrontal cortex of C57 BL/6J (neurotypical phenotype) and C58/J (autistic-like phenotype) mice. n= 20 neurons per strain, 5 neurons per mouse. Kolmogorov-Smirnov test *p<0.05.

**Supplementary Figure 4.**

**Classification of dendritic spines by shape type in the prefrontal cortex of C58/J mice.** Dendritic spines were categorized depending on their length into filopodia-like or non-filopodia-like, and by their width, as mushroom head-like or non-mushroom head-like, in total (a,b), apical (c,d), and basal (e,f) dendrites of pyramidal neurons in the prefrontal cortex of C57 BL/6J (neurotypical phenotype) and C58/J (autistic-like phenotype) mice. Results are expressed as mean ± SD; n= 20 neurons per strain, 5 neurons per mouse. t-test with Holm–Bonferroni correction: *p<0.05.

**Supplementary Figure 5.**

**Classification of dendritic spines by shape based on Risher’s method in the hippocampus and the prefrontal cortex of C58/J mice.** Categorization of dendritic spines as filopodia (Filo), long-thin, thin, stubby (Stub), mushroom with a medium head (Mush M), mushroom with a wide head (Mush W), and branched (Branch) based on Risher’s method (a). Arrowheads are pointing to each shape type. Dendritic spines were classified according to their shape in total, apical, and basal dendrites of pyramidal neurons in the hippocampus (b-d) and the prefrontal cortex (e-g) of C57 BL/6J (neurotypical phenotype) and C58/J (autistic-like phenotype) mice. Results are expressed as mean ± SD; n= 20 neurons per strain, 5 neurons per mouse. t-test with Holm–Bonferroni correction: *p<0.05.

**Supplementary Figure 6.**

**Clustering of dendritic spines by length and width dimensions in the prefrontal cortex of C58/J mice.** Distribution of dendritic spines in three main clusters using the K-means algorithm. Clusters were defined as *small*, *long*, and *wide* spines, according to their length and width parameters (a). Number of dendritic spines grouped in each cluster, in total (b), apical (c), and basal (d) dendrites of pyramidal neurons in the prefrontal cortex of C57 BL/6J (neurotypical phenotype) and C58/J (autistic-like phenotype) mice. Results are expressed as mean ± SD; n= 20 neurons per strain, 5 neurons per mouse. t-test with Holm–Bonferroni correction: *p<0.05.

**Supplementary Figure 7.**

**BDNF protein content in the hippocampus of C58/J mice.** Western blot of BDNF content in the hippocampus of C57 BL/6J (neurotypical phenotype) and C58/J (autistic-like phenotype) mice (a). Arrowheads indicate the corresponding signals of mature BDNF (m-BDNF) and the precursor protein (pro-BDNF). Semi-quantitative analysis of BDNF densitometry for both strains (b). Results are expressed as mean ± SD; n= 4 mice per strain. t-test: *p<0.05.

**Supplementary Table 1.**

SNPs in the genome of the C58/J autistic-like strain in comparison with the C57 BL/6J neurotypical mice. Data retrieved from The Mouse Phenome Database (MPD).

**Supplementary Table 2.**

GO enrichment analysis of C58/J mice polymorphic genes in the GOnet platform.

**Supplementary Table 3.**

**GO enrichment analysis of C58/J mice polymorphic genes.** C58/J autistic-like strain polymorphic genes shared at least five *biological process* and/or *molecular function* terms associated with cytoskeleton organization, neuronal and synapse function, signaling, central nervous system development, and cognitive processes.

**Supplementary Table 4.**

SNPs in the genome of the C58/J autistic-like strain in comparison with the C57 BL/6J neurotypical mice according to the Sanger4 database (MPD database).
