# Supplementary material for: Changes in the Number and Morphology of Dendritic Spines in the Hippocampus and Prefrontal Cortex of the C58/J Mouse Model of Autism
Source: Front Cell Neurosci. 2021 Sep 20;15:726501. doi: 10.3389/fncel.2021.726501 (PMC8488392; doi:10.3389/fncel.2021.726501)
Supplement: Supplementary file 8 [file Data_Sheet_1.zip › New folder (2)/Sup Table 3.DOCX]

**Supplementary Table 3.**

**GO enrichment analysis of C58/J mice polymorphic genes.** C58/J autistic-like strain polymorphic genes shared at least five *biological process* and/or *molecular function* terms associated with cytoskeleton organization, neuronal and synapse function, signaling, central nervous system development, and cognitive processes.

| **GO Biological process/**  **Molecular function** | **Uniprot ID** | **Gene symbol** | **Gene name** | **Orthologous human gene in SFARI** | **Uniprot ID** | **SFARI score** |
| --- | --- | --- | --- | --- | --- | --- |
| 1, 2, 3, 8, 9, 10, 11, 12, 13, 14, 15, 16, 17, 21, 22, 23 | P97333 | Nrp1 | Neuropilin1 |  |  |  |
| 1, 2, 3, 7, 12, 13, 14, 15, 16, 18, 19, 20, 21, 23 | Q04690 | **NF1** | **Neurofibromin 1** | NF1 | P21359 | 1 |
| 1, 2, 3, 9, 10, 11, 12, 13, 14, 15, 21, 22, 23 | Q91YM2 | Arhgap35 | Rho GTPase activating protein 35 |  |  |  |
| 1, 9, 11, 12, 13, 14, 15, 16, 17, 20, 21, 1* | Q9WV60 | Gsk3b | Glycogen synthase kinase 3 beta |  |  |  |
| 6, 7, 12, 13, 14, 18, 19, 1*, 2*, 3*, 4*, 5* | Q60857 | **SLC6A4** | **Solute carrier family 6 (neurotransmitter transporter, serotonin), member 4** | SLC6A4 | P31645 | 3 |
| 1, 3, 4, 5, 12, 13, 14, 15, 16, 1*, 2*, 3* | Q6ZWQ0 | Syne2 | Spectrin repeat containing, nuclear envelope 2 |  |  |  |
| 1, 4, 5, 9, 11, 12, 13, 14, 15, 16, 19 | Q3V3N7 | Bbs1 | Bardet-Biedl syndrome 1 (human) |  |  |  |
| 1, 4, 5, 9, 10, 11, 12, 18, 19, 1*, 2* | Q9QYR6 | **MAP1A** | **Microtubule-associated protein 1A** | MAP1A | P78559 | 1 |
| 8, 11, 12, 13, 14, 15, 16, 18, 19, 20, 21 | P15209 | **NTRK2** | **Neurotrophic tyrosine kinase, receptor, type 2** | NTRK2 | Q16620 | S |
| 1, 4, 5, 11, 12, 13, 14, 15, 18, 19 | P42859 | Htt | Huntingtin |  |  |  |
| 3, 20, 21, 23, 1*, 2*, 3*, 4*, 5*, 6* | Q9QY06 | **MYO9B** | **Myosin IXb** | MYO9B | Q13459 | 2 |
| 1, 2, 3, 8, 12, 21, 23, 1*, 2*, 3* | Q8C0T5 | **Sipa1\|1** | **Signal-induced proliferation-associated 1 like 1** |  |  |  |
| 1, 2, 3, 13, 14, 15, 20, 21, 22, 23 | P05480 | **Src** | **Rous sarcoma oncogene** |  |  |  |
| 9, 11, 12, 13, 14, 15, 18, 19 | Q9CPW0 | **CNTNAP2** | **Contactin associated protein-like 2** | CNTNAP2 | Q9UHC6 | 2S |
| 13, 14, 15, 16, 18, 19, 20, 21 | Q9Z1B3 | **PLCB1** | **Phospholipase C, beta 1** | PLCB1 | Q9NQ66 | 2 |
| 1, 2, 3, 21, 22, 1*, 2*, 3* | P26039 | Tln1 | Talin1 |  |  |  |
| 1, 2, 3, 8, 12, 4*, 5*, 6* | Q8R071 | Itpka | Inositol 1,4,5-trisphosphate 3-kinase A |  |  |  |
| 1, 2, 3, 21, 1*, 2*, 3* | Q62468 | **VIL1** | **Villin 1** | VIL1 | P09327 | 2 |
| 8, 9, 11, 12, 13, 14, 19 | Q9R0K7 | **ATP2B2** | **ATPase, Ca++ transporting, plasma membrane 2** | ATP2B2 | Q01814 | 2 |
| 1, 4, 5, 1*, 4*, 5*, 6* | Q61771 | Kif3b | Kinesin family member 3B |  |  |  |
| 1, 2, 3, 12, 1*, 2*, 3* | O88398 | Avil | Advillin |  |  |  |
| 1, 2, 3, 21, 1*, 2*, 3* | O70373 | Xirp1 | Xin actin-binding repeat containing 1 |  |  |  |
| 1, 2, 3, 20, 23, 4*, 5* | Q8BKH7 | Mapkap1 | Mitogen-activated protein kinase associated protein 1 |  |  |  |
| 8, 12, 21, 23, 4*, 5*, 6* | Q8CHT1 | Ngef | Neuronal guanine nucleotide exchange factor |  |  |  |
| 8, 9, 10, 11, 12, 17, 19 | Q8C110 | Slitrk6 | SLIT and NTRK-like family, member 6 |  |  |  |
| 9, 10, 11, 12, 13, 14, 15 | Q8K0S5 | Rtn4rl1 | Reticulon 4 receptor-like 1 |  |  |  |
| 9, 10, 11, 12, 13, 17, 1* | P11499 | Hsp90ab1 | Heat shock protein 90 alpha (cytosolic), class B member 1 |  |  |  |
| 4, 5, 13, 14, 15, 21 | Q0HA38 | Ttc21b | Tetratricopeptide repeat domain 21B |  |  |  |
| 9, 10, 11, 12, 17, 21 | P70206 | Plxna1 | Plexin A1 |  |  |  |
| 1, 2, 3, 17, 21, 1* | Q9R229 | Bmp10 | Bone morphogenetic protein 10 |  |  |  |
| 8, 9, 11, 12, 17, 21 | Q8VI56 | Lrp4 | Low density lipoprotein receptor-related protein 4 |  |  |  |
| 9, 10, 11, 12, 17, 21 | Q9WVB4 | Slit3 | Slit guidance ligand 3 |  |  |  |
| 4, 5, 9, 11, 12, 1* | Q6PD31 | Trak1 | Trafficking protein, kinesin binding 1 |  |  |  |
| 11, 12, 13, 14, 15, 17 | Q9ER74 | Sall1 | Spalt like transcription factor 1 |  |  |  |
| 11, 12, 13, 14, 20, 4* | Q0KK55 | Kndc1 | Kinase non-catalytic C-lobe domain (KIND) containing 1 |  |  |  |
| 9, 10, 11, 12, 13 | Q91ZX7 | **LRP1** | **Low density lipoprotein receptor-related protein 1** | LRP1 | Q07954 | 2 |
| 12, 13, 14, 18, 19 | O08537 | **ESR2** | **Estrogen receptor 2 (beta)** | ESR2 | Q92731 | 3 |
| 9, 10, 11, 12, 17 | Q63955 | Pou4f3 | POU domain, class 4, transcription factor 3 |  |  |  |
| 9, 11, 12, 1*, 2* | Q1EG27 | Myo3b | Myosin IIIB |  |  |  |
| 8, 9, 10, 11, 12 | Q810B9 | Slitrk3 | SLIT and NTRK-like family, member 3 |  |  |  |
| 12, 13, 14, 15, 20 | Q61501 | E2f1 | E2F transcription factor 1 |  |  |  |
| 9, 11, 12, 19, 21 | O55106 | Strn | Striatin, calmodulin binding protein |  |  |  |
| 9, 10, 11, 12, 20 | Q9WTS5 | Tenm2 | Teneurin transmembrane protein 2 |  |  |  |
| 11, 12, 13, 14, 15 | Q03267 | Ikzf1 | IKAROS family zinc finger 1 |  |  |  |
| 13, 14, 18, 19 | Q924A2 | **CIC** | **Capicua transcriptional repressor** | CIC | Q96RK0 | 1 |
| 18, 19, 21 | Q68ED2 | **GRM7** | **Glutamate receptor, metabotropic 7** | GRM7 | Q14831 | 3 |
| 20, 21 | Q9Z1W9 | **STK39** | **Serine/threonine kinase 39** | STK39 | Q9UEW8 | 3 |
| 17 | O88455 | **DHCR7** | **7-dehydrocholesterol reductase** | DHCR7 | Q9UBM7 | 1 |
| 17 | E9Q4F7 | **ANKRD11** | **Ankyrin repeat domain 11** | ANKRD11 | Q6UB99 | 1 |
| 21 | Q5XJE5 | **LEO1** | **Leo1, Paf1/RNA polymerase II complex component** | LEO1 | Q8WVC0 | 2 |
| 21 | Q8R4V4 | **CPZ** | **Carboxypeptidase Z** | CPZ | Q66K79 | 3 |
| Biological process. 1: cytoskeleton organization, 2: actin cytoskeleton organization, 3: actin filament-based process, 4: microtubule-based process, 5: microtubule-based movement, 6: neurotransmitter reuptake, 7:neurotransmitter transport, 8: synapse organization, 9: neuron projection development, 10: axonogenesis, 11: neuron differentiation, 12: neurogenesis, 13: central nervous system development, 14: brain development, 15: forebrain development, 16: cerebral cortex development, 17: developmental growth, 18: learning or memory, 19: behavior, 20: intracellular signal transduction, 21: cell surface receptor signaling pathway, 22: integrin-mediated signaling pathway, 23: regulation of small GTPase mediated signal transduction. Molecular function. 1*: cytoskeletal protein binding, 2*: actin binding, 3*: actin filament binding, 4*: small GTPase binding, 5*: Ras GTPase binding, 6*: Rho GTPase binding. | | | | | | |
